# Interhemispheric auditory connectivity: structure and function related to auditory verbal hallucinations

**DOI:** 10.3389/fnhum.2014.00055

**Published:** 2014-02-11

**Authors:** Saskia Steinmann, Gregor Leicht, Christoph Mulert

**Affiliations:** Psychiatry Neuroimaging Branch, Department of Psychiatry and Psychotherapy, University Medical Center Hamburg-EppendorfHamburg, Germany

**Keywords:** auditory-verbal hallucinations, schizophrenia, structural and functional interhemispheric connectivity, auditory cortex, neuroimaging

## Abstract

Auditory verbal hallucinations (AVH) are one of the most common and most distressing symptoms of schizophrenia. Despite fundamental research, the underlying neurocognitive and neurobiological mechanisms are still a matter of debate. Previous studies suggested that “hearing voices” is associated with a number of factors including local deficits in the left auditory cortex and a disturbed connectivity of frontal and temporoparietal language-related areas. In addition, it is hypothesized that the interhemispheric pathways connecting right and left auditory cortices might be involved in the pathogenesis of AVH. Findings based on Diffusion-Tensor-Imaging (DTI) measurements revealed a remarkable interindividual variability in size and shape of the interhemispheric auditory pathways. Interestingly, schizophrenia patients suffering from AVH exhibited increased fractional anisotropy (FA) in the interhemispheric fibers than non-hallucinating patients. Thus, higher FA-values indicate an increased severity of AVH. Moreover, a dichotic listening (DL) task showed that the interindividual variability in the interhemispheric auditory pathways was reflected in the behavioral outcome: stronger pathways supported a better information transfer and consequently improved speech perception. This detection indicates a specific structure-function relationship, which seems to be interindividually variable. This review focuses on recent findings concerning the structure-function relationship of the interhemispheric pathways in controls, hallucinating and non-hallucinating schizophrenia patients and concludes that changes in the structural and functional connectivity of auditory areas are involved in the pathophysiology of AVH.

## Introduction

Auditory verbal hallucinations (AVH) are one of the most prominent symptoms in schizophrenia (SZ), affecting approximately 70% of patients (Schneider, 1957), a fact reflected in their use as a major diagnostic criterion for the disease (Diagnostic and Statistical Manual IV, 1994). AVH are simply defined as vocal perceptions without an appropriate external stimulus (Woodruff, 2004). They mostly occur as voices (i.e., “hearing voices”) that might comment on or criticize the patient and are therefore thought to reflect dysfunctional auditory processing in speech-relevant brain areas.

Various theoretical models have been proposed regarding the cognitive mechanisms underlying AVH: one currently influential hypothesis is that AVH represent internally-generated speech (i.e., thinking in words) misidentified as coming from outside the self because of defective self-monitoring (Frith, 1995; for review see Waters et al. (2012). This is assumed to result from a dysfunction in the “forward-model” system, the role of which consists in predicting the sensory consequences of actions. Consequently, patients are not in control of their own inner speech (Frith, 2005).

Despite a large body of research in this area, the etiology of AVH remains unknown and the underlying physiological causes are still not fully understood. While structural neuroimaging studies of AVH initially focused on gray matter (GM) abnormalities in the left auditory cortex, more recent studies have instead emphasized the role of changes in fronto-temporal white matter (WM) fiber tracts relevant for speech. Currently, AVH is thought to result not only from focal impairments in single speech-related areas, but also from altered functional connectivity between these and other brain regions. Thus, research on the neurobiology of AVH emphasizes a network view postulating dysfunctional interactions among a range of brain regions and functions.

This review begins with a brief overview of structural and functional neuroimaging findings on AVH. Included are studies published between 1990 and 2013 that have used either electroencephalography (EEG, Berger, 1929) or magnetic resonance imaging (MRI) techniques such as voxel-based morphometry (VBM, Ashburner and Friston, 2000), diffusion-tensor-imaging (DTI, Basser et al., 1994), and functional MRI (fMRI). The main section of the review concentrates on findings regarding interhemispheric connectivity between bilateral auditory cortices.

## Neuroimaging studies on AVH: current state of knowledge

### Structural neuroimaging

Research into the pathophysiology of AVH has boomed in the past two decades thanks to the advent of modern neuroimaging methods based on MRI techniques (e.g., VBM), which enabled researchers to gain initial insights into the brain regions and networks involved in this challenging symptom. One of the most extensively investigated brain regions in this regard is the left temporal lobe, in particular the primary (PAC or Heschl's gyrus), and secondary (SAC) auditory cortex. These cortical areas are known to be crucial for auditory perception and comprehension (Friederici, 2011).

Accordingly, in patients who suffer from AVH reduced gray matter volume (GMV) has been reported in predominantly speech-related brain areas such as the left superior temporal gyrus (STG) (Barta et al., 1990; Shenton et al., 1992), Heschl's gyrus (Gaser et al., 2004; Nenadic et al., 2010), left middle temporal gyrus (MTG), and left temporo-parietal regions (for a detailed review see Allen et al., 2008). A recent meta-analysis of VBM studies that included a total of 438 patients (307 with AVHs) confirmed a significant correlation between the extent of volume loss in the Heschl's gyrus and severity of AVH (Modinos et al., 2013). Marginal GMV reductions were also noted in the right STG, thus suggesting that both the left and right STG are involved in the structural pathology of AVH.

Further, evidence so far suggests that GMV reductions in areas other than sensory cortices also contribute to hallucinatory experiences. Such areas include the insula bilaterally, left amygdala, left inferior frontal gyrus (IFG), parahippocampal gyrus, anterior and posterior cingulate cortex (ACC and PCC), thalamus, cerebellum, and precuneus (Allen et al., 2008; García-Martí et al., 2012). More specifically, the insula and the amygdala—key regions for emotional regulation—are postulated to be involved in the commonly observed emotional load of AVH (Alba-Ferrara et al., 2012), while dysfunction of the PCC—known to constitute a basic component in generating a model of the self—is responsible for defective self-referential processing (Northoff and Bermpohl, 2004).

In brief, changes in the auditory cortex and other language-related brain areas are the most consistent and replicated findings in structural imaging studies of AVH. However, other cortical regions and the limbic system must also be taken into account, especially for neuropsychological models.

### Functional neuroimaging

Findings of the aforementioned structural studies are supported by several functional imaging studies demonstrating that “hearing voices” involves similar brain regions as normal speech perception and production. For example, acute experience of AVH has been associated with increased activation in bilateral auditory cortices, including the Heschl's gyrus (Dierks et al., 1999; Lennox et al., 2000; van de Ven et al., 2005) and Broca's area (McGuire et al., 1993). Moreover, Shergill et al. (2000a) associated AVH with activations in the inferior frontal cortex and insula, ACC, MTG bilaterally (though somewhat lateralized to the right), the right thalamus and inferior colliculus, as well as the left hippocampus and parahippocampal cortex. Thus, regions not directly related to language such as the left hippocampus/parahippocampus—an area thought to be involved in verbal memory—also exhibit increased activation during AVH. Based on this finding, it has been suggested that aberrant retrieval of verbal memories might be one of the factors associated with the involuntary emergence of AVH (Jardri et al., 2011).

In addition, Woodruff et al. (1997) also reported that hallucinating patients demonstrate greater activity in the right than left SAC while listening to speech, i.e., they exhibit a reversed pattern of the typical (left-lateralized) auditory cortex response; this finding was interpreted as a possible consequence of altered interhemispheric connectivity (Woodruff, 2004). Interestingly, a current meta-analysis of dichotic listening studies has suggested reduced left-hemispheric language lateralization as a strong trait marker for the occurrence of AVH within the SZ population (Ocklenburg et al., 2013).

Further, an fMRI study looking into the time course of brain activation immediately prior to AVH onset described activations in the left anterior insula and right MTG, as well as deactivation in the ACC and parahippocampal gyrus (Hoffman et al., 2008). Notably, two previous studies had also reported increased activation in the right MTG prior to the onset of AVH. It is hypothesized that these neural events trigger hallucinatory experiences or predispose patients to them (Lennox et al., 1999; Shergill et al., 2004).

In another study, fMRI was applied to identify neural activation patterns during tasks tapping into verbal self-monitoring processes in patients predisposed to AVH, while they were imagining external auditory speech; attenuated activation was found in the posterior cerebellum, hippocampi, lenticular nuclei bilaterally, the right thalamus, MTG and STG, and left nucleus accumbens (Shergill et al., 2000b). These structures have been suggested to serve as comparators in models of cognitive self-monitoring (Gray et al., 1991), and are also associated with memory retrieval (Henke et al., 1999). Thus, reduced activation during auditory verbal imagery might be related to defective auditory-verbal recall in patients.

Finally, a coordinate-based meta-analysis of 10 fMRI studies looking into the brain areas that are predominantly activated during the experience of AVH provided support for the aforementioned findings and also identified a more widely distributed fronto-temporal language-related network involving the left MTG and STG, Broca's area, bilateral frontal operculum, bilateral anterior insula, left precentral gyrus, and left supramarginal gyrus (Jardri et al., 2011).

### Functional and structural connectivity studies

As mentioned earlier, one current model of AVH suggests that they arise from a disability of patients to recognize self-generated inner speech (Frith, 1995). This model postulates a disturbance in the mechanisms that serve to predict the outcome of inner speech (forward model). Many models of AVH conceptualize the symptom in terms of a failure to integrate information regarding inner speech, generated in the frontal cortex, with sensory processing in temporal and parietal regions (Friston and Frith, 1995; Frith, 2005). There is increasing experimental support for this hypothesis: for example, reduced fronto-temporal connectivity has been observed in SZ patients with AVH during a sentence-completion task (Lawrie et al., 2002), external speech processing (Mechelli et al., 2007), or speaking aloud (Ford et al., 2002). More recently, a resting-state fMRI study demonstrated reduced functional connectivity between the left temporo-parietal junction—a critical node for speech perception and AVH genesis (Hoffman et al., 2003)—and the right frontal homotope of Broca's area—associated with speech production in hallucinating patients (Vercammen et al., 2010). In addition, more severe AVH were associated with reduced neural synchrony between the left temporo-parietal junction and bilateral ACC as well as bilateral amygdala; again, these results suggest a dysfunctional interaction among brain areas involved in self-referential processing, attribution of agency, and attentional control (Vercammen et al., 2010).

The introduction of Diffusion Tensor Imaging (DTI) has made examination of specific WM fiber tract characteristics (size, shape, spatial organization) possible (Basser et al., 1994). DTI methods exploit properties of molecular diffusion and characterization of the diffusion tensor to infer the orientation and integrity of WM tracts in the brain. Diffusion is subjected to certain restrictions such as the type of cellular tissue, or the presence of myelin sheaths or cell membranes. As a result, the spatial orientation of fibers can be inferred from its main direction (Mori et al., 1999; Nucifora et al., 2007). The most commonly used metric of diffusion anisotropy is “fractional anisotropy” (FA), a value which varies on a scale from 0 (isotropic) to 1 (anisotropic) (Pierpaoli and Basser, 1996), whereby higher values indicate more parallel alignment of cell membranes or other bio-barriers. In clinical studies, FA values are routinely used as an index of WM integrity and are sensitive to WM deterioration in neurodegenerative and psychiatric disorders (Zhang et al., 2007; Mulert et al., 2011).

In the context of AVH, an early DTI study reported increased FA values of the arcuate fasciculus in hallucinating patients compared to non-hallucinating patients and healthy controls (HC). Since the arcuate fasciculus constitutes the major fiber tract connecting inferior frontal and temporo-parietal areas involved in language perception and production, the above finding indicates stronger connectivity between frontal speech production (i.e., Broca's area) and temporal speech perception areas (i.e., Wernicke's area) in hallucinating patients (Hubl et al., 2004). Interestingly, increased FA values of the arcuate fasciculus have been also demonstrated in non-psychotic subjects suffering from AVH (de Weijer et al., 2011). This implies that alterations of the arcuate fasciculus might be specifically associated with the symptom of AVH rather than SZ.

Another DTI study investigated the arcuate fasciculus in more detail by dividing it into three segments: anterior indirect, posterior indirect and long direct segment (Catani et al., 2011). The anterior indirect segment connects fronto-parietal regions (i.e., Broca's area with Geschwind's area) and appears to relate to speech articulation, whereas the posterior indirect segment connects temporo-parietal regions (i.e., Wernicke's area with Geschwind's area) and is related to speech comprehension (Duffau, 2008). The long direct segment represents the classical arcuate fasciculus. Results showed bilaterally significant reduced FA values in patients, which was however limited to the long direct and the posterior indirect segment, projecting both to the posterior temporal auditory regions associated with speech perception. Interestingly, FA values were lowest in patients with AVH.

However, one earlier study also reported increased FA values in the lateral part of the superior longitudinal fasciculi (SLF) and the ACC in hallucinating patients compared to non-hallucinating patients, while in comparison to healthy controls the FA values were still lower even in the patients who were most prone to AVH (Shergill et al., 2007). The authors suggested that the severity of these changes may vary with the characteristics of symptoms. Further, Makris et al. (2010) demonstrated a significant positive correlation between WM volume in anterior callosal, cingulate and temporal deep WM regions on one hand and positive symptoms, such as AVH or delusions, on the other.

## Interhemispheric auditory connectivity in healthy controls (HC)

**Figure 1:**
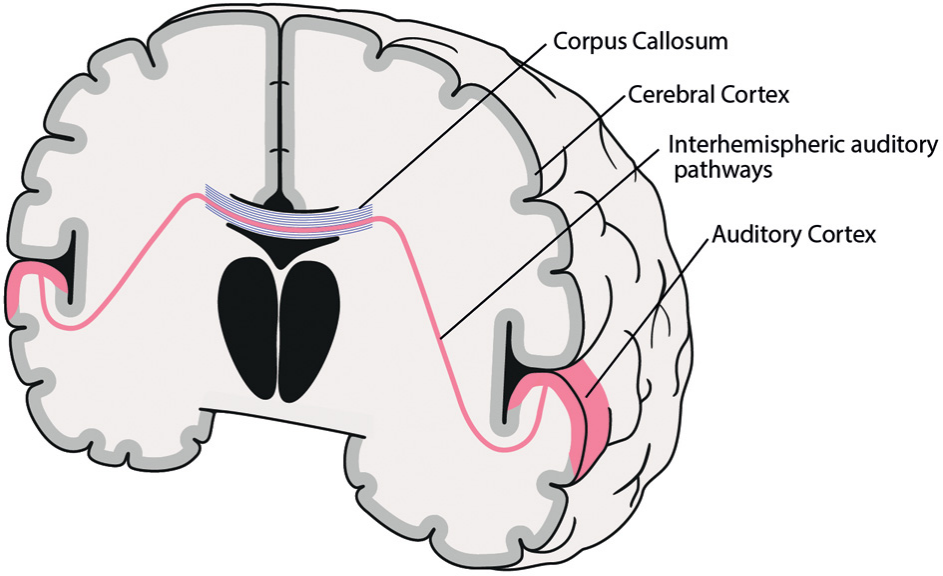
**Coronal schematic illustration of the interhemispheric auditory pathways connecting right and left auditory cortices**. Corpus Callosum (blue), interhemispheric auditory pathways connecting right and left auditory cortices (red).

While all of the factors mentioned above may be involved in the genesis of AVH, the following part will concentrate on the role of interhemispheric pathways between right and left auditory cortices (see Figure 1).

### Interhemispheric auditory pathway structure

Interhemispheric interaction is primarily subserved by the brain's commissural system, including the corpus callosum (CC), anterior commissure, posterior commissure, cerebellar commissures, and interthalamic adhesions (Hoptman and Davidson, 1994). The CC is by far the largest WM structure in the human brain, containing more than 300 million transcallosal fibers (Hofer and Frahm, 2006), which are spatially ordered according to their point of origin in the cortex (Schmahmann and Pandya, 2006). So, for example, fibers connecting frontal areas progress through the most anterior part of the CC (genu), while bilateral temporal, parietal or occipital cortices are connected through the posterior parts (isthmus, splenium) of the CC. Thus, interhemispheric interaction between PAC and SAC—both located at the STG—is primarily mediated by interhemispheric auditory pathways running through the posterior third of the CC (isthmus, splenium) (Aboitiz et al., 1992; Bamiou et al., 2007). Moreover, the diameter of various CC subregions varies widely, ranging from small (0.4–1 μm) to large (>3 μm). Interestingly, interhemispheric auditory pathways are among the densest and present the largest fiber diameters (>3 μm), and thus possess fast conduction velocities, which are assumed to be necessary for fast bilateral interaction, e.g., for sound localization in space (Aboitiz et al., 1992).

### Interhemispheric auditory pathway function

The functional relevance of interhemispheric auditory fibers was investigated in an event-related potential study that examined interhemispheric interaction of prosodic and syntactic information during speech comprehension in patients with lesions of the posterior third of the CC (Friederici et al., 2007). Results demonstrated that the posterior third of the CC—where interhemispheric auditory fibers cross—is crucial for the integration of prosodic information (right hemisphere) with syntactic information (left hemisphere), which is in turn a prerequisite for spoken language comprehension. In the study by Friederici et al., participants performed a comprehension task that required successful integration of prosodic and syntactic information. Patients with lesions of the posterior part of the CC exhibited an absence of the N400, an event-related potential assumed, in this context, to reflect successful interaction between right and left auditory areas. Thus, failure to produce the N400 potential in patients was interpreted by the authors as evidence for a disconnection of interhemispheric auditory pathways that leads to disrupted information flow among regions associated with lateralized language functions.

Similar results were obtained in studies using the dichotic listening (DL) task. In this paradigm, two different consonant-vocal (CV)-syllables such as “ba” or “ga” are paired and presented simultaneously, one in the right ear (RE) and another one in the left ear (LE). A typical finding across studies in healthy right-handed individuals is the so-called Right Ear Advantage (REA), i.e., subjects report the syllable presented to the RE with better accuracy than the one presented to the LE (Kimura, 1961; Hugdahl, 2003). The REA reflects the dominance of the left hemisphere in speech perception as well as the fact that, under DL conditions, auditory information is transferred predominantly through contralateral auditory pathways, while ipsilateral pathways are inhibited (Brancucci et al., 2004). According to the “callosal relay model,” the CV-syllable arriving from the LE to the non-dominant right hemisphere requires additional interhemispheric transfer across the CC before it can be efficiently processed in the dominant left hemisphere (Kimura, 1967; Bryden, 1988; Hugdahl, 1995). In this regard, the LE-report serves as an index of interhemispheric transfer.

Pollmann et al. (2002) reported that patients with lesions in the posterior part of the CC did not report any syllables presented to the LE, showing an almost perfect REA. Moreover, studies in patients with multiple sclerosis have revealed a reduced LE report, which correlated with the extent of atrophy of the posterior part of the CC (Barkhof et al., 1998; Pelletier et al., 2001; Gadea et al., 2002). These results indicate that the perception of CV-syllables presented to the LE depends on the integrity of interhemispheric auditory pathways.

In summary, interhemispheric auditory pathways appear to play a crucial role in healthy auditory processing and speech comprehension, such that impaired interhemispheric connectivity leads to disrupted information flow between homologous auditory areas and, consequently, to disturbance of language-related processes.

### Structure-behavior relationship of the interhemispheric auditory pathways

Recent DTI and tractography studies have revealed striking interindividual variability in the size and shape of interhemispheric auditory pathways in the general population (Hofer and Frahm, 2006; Dougherty et al., 2007; Westerhausen et al., 2009). For instance, Westerhausen et al. (2009) investigated the functional relevance of callosal fiber tract interindividual variability in 17 HC with probabilistic DTI tractography. The authors identified the callosal substrate connecting the posterior STG with the left and right Heschl's gyrus and subsequently correlated structural data with individual performance on a DL task of CV-syllables. Results showed that interindividual variation of callosal topography was reflected in behavioral performance: individuals with stronger anatomical pathways between bilateral auditory cortices exhibited better information processing and, consequently, improved perception of the CV-syllable presented to the LE.

Contrary findings were published by Dougherty et al. (2007), who investigated interhemispheric callosal fibers in 55 children with a wide range of reading ability using DTI tractography. Their results showed that phonological awareness (a key factor in reading acquisition) was positively correlated with radial diffusivity of the interhemispheric pathways connecting temporal lobes. Based on these findings, the authors argued that good readers presumably have a higher proportion of large axons but also fewer callosal connections in total and, hence, reduced interhemispheric connectivity.

To the best of our knowledge, no study has so far concurrently investigated the structural integrity of interhemispheric auditory pathways along with the functional activity of the regions they connect. Nevertheless, the above findings indicate a large interindividual variability regarding the relationship between interhemispheric fiber structure and auditory task performance. In addition, several studies have suggested that dysfunctional connectivity between the left and right auditory cortex may lead to deficits in auditory processing (Barkhof et al., 1998; McKay et al., 2000; Pelletier et al., 2001; Gadea et al., 2002; Pollmann et al., 2002). Interestingly, it has been suggested that alterations in the interhemispheric connectivity attended by a disturbed interaction may be associated with unusual auditory perceptions and/ or AVH in predisposed individuals (Rossell et al., 2001; Woodruff, 2004; Rossell and Boundy, 2005).

## Altered structural and functional interhemispheric auditory connectivity and AVH

With use of novel neuroimaging measures of WM structure, altered interhemispheric connectivity between the right and left auditory cortex has been observed in hallucinating patients (see Table 1).

**Table 1:** **Characteristics of included studies investigating the interhemispheric connectivity related to AVH**.

**Study**	**Imaging modality**	**Participants**	**Diagnostic criteria and symptom ratings**	**Paradigm**	**Region of interest**	**Results**
Hubl et al., 2004	DTI	AVH patients (*n* = 13)	ICD-10, PANSS, CGI	No task	CC	Sign. increased FA values in the posterior third of the CC in AVH patients
		Non-AVH patients (*n* = 13)				
		Healthy controls (*n* = 13)				
Mulert et al., 2011	DTI	First episode AVH patients (*n* = 5)	DSM IV, SAPS	No task	Interhemispheric auditory pathways	Sign. increased FA values of the interhemispheric auditory pathways in AVH patients
		First episode Non-AVH patients (*n* = 5)				
		Healthy controls (n=10)				
Knöchel et al., 2012	Structural MRI and DTI	Chronic patients (*n* = 16)	DSM-IV, PANSS, RHS	No task	CC	Reduced FA values and increased MD values of the whole CC, indicating decreased compactness and increased intercellular space
		First degree relatives (*n* = 16)				
		Healthy controls (*n* = 15)				
Gavrilescu et al., 2010	fMRI	AVH patients (*n* = 14)	DSM IV, PANSS	Passive listening to words	Functional interhemispheric auditory connectivity between both auditory cortices	Sign. reduced functional interhemispheric connectivity in AVH patients
		Non-AVH patients (*n* = 13)				
		Healthy controls (*n* = 6)				
Henshall et al., 2012	EEG	AVH patients (*n* = 19)	DSM IV, PANSS	Monaurally presentation of pure tones and single-syllable words	Interhemispheric transfer time (IHTT) between both auditory cortices	Sign. different IHTT in the word condition across the 3 groups: highest IHTT in AVH patients, indicating transcallosal dysfunctions
		Non-AVH patients (*n* = 17)				
		Healthy controls (*n* = 17)				
Mulert et al., 2012	EEG	Chronic patients (*n* = 18)	DSM-IV, SAPS	40-Hz ASSR	Interhemispheric auditory connectivity (lagged phase synchronization)	Sign. reduced long-range synchrony of gamma oscillations between right and left PAC in SZ patients
		Healthy controls (*n* = 16)				
McKay et al., 2000	Auditory brainstem responses (ABR)	AVH patients (*n* = 22)	DSM-III-R, MUPS	Battery of nine standard audio-logical tests	Behavior	Sign. poorer performance, interpreted either as altered right auditory cortex functions and/or as interhemispheric pathway deficits
		Non-AVH patients (*n* = 16)				
		Healthy controls (*n* = 22)				

*AVH patients, schizophrenic patients with hallucinations; Non-AVH patients, patients without hallucinations; ICD, International Classification of Diseases; PANSS, Positive and Negative Symptom Scale; CGI, Clinical Global Impression Scale; SAPS, Scale for the Assessment of Positive Symptoms; RHS, Revised Hallucination Scale; DSM, Diagnostic and Statistical Manual; MUPS, Mental Health Research Institute Unusual Perceptions Schedule; ASSR, Auditory-Steady-State-Response; PAC, Primary Auditory Cortex; CC, Corpus Callosum*.

### Findings of increased interhemispheric connectivity

In addition to the aforementioned finding of increased FA values in the arcuate fasciculus in patients with schizophrenia, Hubl et al. (2004) also reported increased FA values in the posterior third of the CC in 13 acutely ill patients with SZ and frequent AVH compared to 13 acutely patients who had never experienced any hallucinations in their life and HC. When considered as one single group, patients with SZ did not differ from HC regarding FA values. Similar results were reported by a DTI tractography study investigating interhemispheric auditory pathways in relation to AVH vulnerability in 10 patients with first-episode SZ and 10 HC (Mulert et al., 2011). Again, results indicated no differences between SZ patients and HC. However, when the SZ group was split into two subgroups according to the presence or absence of AVH (each 5 patients), significantly increased FA values were found in hallucinating patients in comparison to non-AVH patients and HC.

Both of the above studies suggest that enhanced transcallosal interhemispheric interaction is associated with increased severity of AVH. This conclusion is consistent with findings of neuropsychological studies that have demonstrated reduced REA or even a left ear advantage (LEA) in the DL task in patients with SZ, especially those suffering from AVH (Green et al., 1994; Bruder et al., 1995; Rossell and Boundy, 2005; Hugdahl et al., 2008): Green et al. (2008) reported complete absence of a REA in hallucinating patients, indicating an almost perfect LEA. Moreover, Hugdahl et al. (2008) found a negative correlation between the ability to report the RE stimulus and BPRS (Brief Psychiatric Rating Scale) hallucination scores in 87 right-handed SZ patients, using a DL-task of CV-syllables.

Hugdahl et al. interpreted their finding of reduced REA as reflecting disturbed left temporal lobe function. However, in light of connectivity studies presented above, an alternative explanation for the above findings might be a reduced hemispheric asymmetry in patients, possibly resulting from increased interhemispheric information transfer between the right and left auditory cortex: taking into account the suggestion by Westerhausen et al. (2009) that stronger anatomical interhemispheric pathways between auditory cortices lead to improved LE-report, as well as findings by Hubl et al. (2004) and Mulert et al. (2011) indicating stronger interhemispheric connectivity in hallucinating patients, one might expect an increased LE-report in patients suffering from AVH.

### Findings of decreased interhemispheric connectivity

The above findings of increased interhemispheric connectivity in patients with SZ have not always been replicated: opposite results were reported by Gavrilescu et al. (2010), who used fMRI to examine interhemispheric connectivity between bilateral PAC and SAC cortices in 14 SZ patients currently experiencing AVH, 13 patients without any lifetime history of AVH, and 16 HC. Hallucinating patients exhibited significantly reduced interhemispheric connectivity between auditory cortices compared to patients without AVH and HC, conceivably reflecting structural disconnection. No significant differences were found between patients without any history of AVH and HC.

A recent DTI study also reported reduced CC volume in 16 chronic SZ patients and their first-degree relatives, which was more pronounced in the posterior genu, isthmus, and splenium (Knöchel et al., 2012). Moreover, decreased FA values in the isthmus and increased mean diffusivity (MD) values of the whole CC and isthmus were observed in patients and their unaffected relatives, indicating decreased fiber density and increased intercellular space. Notably, severity of AVH was associated with volumetric decrease of the CC and reduced fiber integrity.

Thus, both of the above studies suggest reduced interhemispheric connectivity as the basis of AVH, quite in contrast to aforementioned findings of enhanced interhemispheric auditory pathways in hallucinating patients. Non-neuroimaging support for reduced interhemispheric connectivity is supplied by a study by McKay et al. (2000), who employed a battery of nine standard audiological tests to study central auditory processing in 22 currently hallucinating patients with SZ, 16 non-hallucinating patients, and 22 HC. Hallucinating patients exhibited significantly poorer performance in a frequency tone pattern test and a staggered spondaic words test compared to both patients without AVH and HC, suggesting either a functional disturbance in the right auditory cortex and/or interhemispheric communication deficits.

An EEG study also examined interhemispheric functional connectivity between bilateral auditory cortices in SZ patients with and without AVH and HC; the measure of interest was interhemispheric transfer time (IHTT) during monaural presentation of pure tones or single-syllable words (Henshall et al., 2012). IHTT was calculated based on temporal information obtained from the latency of the auditory N1 evoked potential. No differences were found for pure tones, but IHTT differed significantly in the word condition across the 3 groups: Hallucinating patients exhibited the highest IHTT values, whereas in HC IHTT was close to zero, and in non-AVH patients it had a negative value. The authors argued that different IHTT might arise from either transcallosal dysfunction in hallucinating patients or from abnormal cerebral lateralization in non-AVH patients.

Additional evidence for disturbed interhemispheric connectivity in SZ has been provided by an EEG study that investigated the functional connectivity between right and left primary and secondary auditory cortices in 18 chronic SZ patients and 16 HC, using phase synchronization in the context of a 40 Hz auditory steady-state response task (ASSR) (Mulert et al., 2012). The major finding was reduced long-range synchrony of gamma oscillations between the right and left primary auditory cortex (PAC) in patients, but not between bilateral secondary auditory cortices. Notably, reduced interhemispheric gamma synchrony was associated with auditory hallucination scores, suggesting that disturbed interhemispheric connectivity between bilateral PAC in the gamma band might be relevant for the emergence of AVH.

### Mixed findings and the need of further research

Modern neuroimaging techniques have provided us with initial insights into what is happening in the brain of hallucinating patients. Volumetric and functional studies consistently report structural and functional disturbances predominantly in speech-related brain areas, but also in limbic and non-sensory regions. The existing literature does not support the hypothesis of increased interhemispheric connectivity in patients with SZ in general, i.e., when the present or absence of AVH is not taken into account. SZ is generally regarded as a disorder of dysfunctional connectivity and disturbed integration among neuronal systems, reflected in volume reductions affecting both gray matter and WM regions (Friston and Frith, 1995; Csernansky and Cronenwett, 2008). In hallucinating patients however, more recent studies using DTI have revealed both increased and decreased interhemispheric connectivity between the right and left auditory cortex (Hubl et al., 2004; Mulert et al., 2011; Knöchel et al., 2012).

On the one hand, findings of more prominent interhemispheric pathways (Hubl et al., 2004; Mulert et al., 2011) support the theory of “hyperconnectivity” in hallucinating patients, as suggested by John et al. (2008). These authors used MRI to investigate the whole CC and its subregions and observed in SZ patients a significant volume increase in the anterior truncus, which possibly comprises interhemispheric fibers that connect temporal association cortices bilaterally. Accordingly, they suggested that an “abnormal functional hyperconnection” might be involved in the emergence of positive SZ symptoms.

According to the above it might be postulated that, although increased interhemispheric auditory connectivity in healthy participants appears to improve speech perception, it might in contrast be disadvantageous for SZ patients, leading to increased severity of “hearing voices.” Thus, one assumption is that stronger interhemispheric connectivity—possibly in combination with further disturbances in the STG or in fronto-temporal connections—might contribute to the emergence of AVH in predisposed individuals (Mulert et al., 2011).

This idea fits nicely to a recent MRI study examining the midsagittal cross-sectional area of the CC with regard to tinnitus (Diesch et al., 2012), which is—similar to AVH—an auditory phantom percept that is experienced in the absence of an external auditory stimulus. Interestingly, also tinnitus patients demonstrated larger volume of the splenium (the CC area where the interhemispheric auditory pathways cross). Moreover, subjective intrusiveness of tinnitus was correlated positively with the size of the majority of the CC subregions, including the posterior parts. Assuming that it is hypersynchronous firing of neurons in the auditory cortex that represents the neurophysiological correlate of tinnitus (Eggermont, 2007), Diesch and colleagues suggested that stronger interhemispheric auditory pathways may facilitate the development and persistence of a positive feedback loop between tinnitus generators located in both hemispheres. Thus, it could be hypothesized that stronger interhemispheric connectivity between auditory areas represent one part of the underlying pathophysiology involved in auditory phantom precepts such as AVH or tinnitus.

On the other hand, one DTI study (Knöchel et al., 2012) and one fMRI-study (Gavrilescu et al., 2010) have demonstrated reduced interhemispheric connectivity in patients, supporting the “disconnectivity hypothesis” of SZ (Friston and Frith, 1995). Moreover, EEG findings of longer IHTT (Henshall et al., 2012) or decreased gamma synchrony (Mulert et al., 2012) indicate reduced interhemispheric connectivity in hallucinating patients. Gavrilescu et al. (2010) have suggested that such disrupted integration of information between the right and left auditory cortex gives rise to aberrant auditory processing, which might be experienced as “external voices.” Since primary, secondary, and auditory association cortices are specialized for different integrative speech functions, it is conceivable that the above disturbances might lead to extensive deficits ranging from basic auditory processing to higher-order language processing.

Altogether, at present there is insufficient neuroimaging evidence to fully depict the structural and functional contribution of interhemispheric pathways to AVH, because of the wide discrepancy of findings. Current evidence suggests some involvement of interhemispheric pathways in the pathophysiology of AVH, but their specific role remains ambiguous. However, reviewed studies all have one finding in common: significant differences concerning FA values of interhemispheric pathways are only found when comparing hallucinating patients to patients with no lifetime experience of AVH or HC. Patients without AVH do not differ from HC, suggesting that interhemispheric connectivity disturbances are specific to the presence of AVH.

The observed inconsistencies among studies might be due to methodological differences. Therefore, and because of the small number of existing studies, it is difficult to draw generalizable conclusions, especially when subgroups (e.g., AVH vs. non-AVH patients) are investigated. More specifically, phenomenological features of hallucinating experiences (such as loudness, perceived source—external vs. internal-, number and/or overlap of voices, type, etc.) are not reported in detail, such that their effect on findings cannot be considered. Most commonly, neuroimaging research has so far tended to neglect phenomenological features, making only a crude distinction between hallucinating and non-hallucinating patients. Similarly, other important variables such as age, duration of illness, medication, and clinical status (first-episode, acute exacerbation, chronicity) might affect results and need to be considered in future studies. A clearly defined clinical diagnosis and a careful phenomenological assessment are essential in order to elucidate the association between specific hallucinatory characteristics and functional/structural deficits and also to enhance comparability of findings. Thus, one next step to progress in the understanding of AVH might be a longitudinal approach to study developmental trends toward an increase or decrease of the interhemispheric fibers' integrity across the clinical status (prodromal, first-episode, and chronicity patients with AVH, ideally matched with non-hallucinating patients).

One potential approach for the investigation of functional connectivity between both auditory cortices might be resting-state (rs) fMRI using independent component analysis (ICA). This approach has been used several times (Mantini et al., 2007; Doucet et al., 2011; Arbabshirani et al., 2013; Storti et al., 2013) identifying commonly a bilateral auditory component covering the primary and secondary auditory areas within Heschl's gyrus and STG. Although there have been ICA/rs-fMRI studies focused on SZ (Mannell et al., 2010; Yu et al., 2013), the topic of AVH has not yet been addressed. However, in the broader context of auditory phantom precepts, there have been studies of tinnitus (Kim et al., 2012; Maudoux et al., 2012; Davies et al., 2013, for review see Husain and Schmidt, 2014) that demonstrated—among others—altered functional interhemispheric connectivity between bilateral auditory cortices. Thus, rs-fMRI could be useful to investigate possible alterations in resting-state neuronal activity between auditory and non-auditory networks in hallucinating patients.

So far, there have been no multimodal studies that have conjointly investigated structural aspects of interhemispheric pathways and functional activations of the regions they connect. Such a framework—e.g., by combining simultaneous EEG-fMRI (with high temporal and spatial resolution) and DTI—that interfaces functional and structural neuroimaging methods would facilitate progress in understanding the precise contribution of the interhemispheric auditory connectivity to AVH and could provide new insights into the involved brain networks. Further, this approach would help clarify whether interhemispheric fiber tracts are reduced or increased in integrity or amount, or possibly misdirected.

## Conclusion

In conclusion, the present review provides an up-to-date summary of findings from studies examining the role of interhemispheric auditory connectivity in the pathophysiology of AVH across different modalities (brain structure, function, and behavior) and populations (HC, SZ patients with and without AVH). The most consistent findings across studies are functional and structural interhemispheric connectivity disturbances in hallucinating patients compared to non-hallucinating patients and HC. Future research on AVH needs to address methodological limitations of existing studies such as the failure to take into account phenomenological features and clinical status. Moreover, studies investigating both structural and functional aspects of interhemispheric connectivity and their relationship are warranted to improve our understanding of the precise contribution of interhemispheric fibers to AVH and to gain new insights into the neurophysiological mechanisms underlying AVH.

### Conflict of interest statement

The authors declare that the research was conducted in the absence of any commercial or financial relationships that could be construed as a potential conflict of interest.
